# Knee cartilage loss in symptomatic knee osteoarthritis over 4.5 years

**DOI:** 10.1186/ar1962

**Published:** 2006-05-16

**Authors:** Anita E Wluka, Andrew Forbes, Yuanyuan Wang, Fahad Hanna, Graeme Jones, Flavia M Cicuttini

**Affiliations:** 1 Department of Epidemiology and Preventive Medicine, Monash University – Central and Eastern Clinical School, Alfred Hospital, Commercial Road, Melbourne, VIC 3004, Australia; 2 Baker Heart Research Institute, 75 Commercial Road, Prahran VIC 3181 Australia; 3 Menzies Research Institute, University of Tasmania, Hobart, 17 Liverpool St, Hobart TAS 7000, Australia

## Abstract

The objective of this study was to describe the rate of change in knee cartilage volume over 4.5 years in subjects with symptomatic knee osteoarthritis (OA) and to determine factors associated with cartilage loss. One hundred and five subjects were eligible for this longitudinal study. Subjects' tibial cartilage volume was assessed by magnetic resonance imaging (MRI) at baseline, at 2 years and at 4.5 years. Of 105 subjects, 78 (74%) completed the study. The annual percentage losses of medial and lateral tibial cartilage over 4.5 years were 3.7 ± 4.7% (mean ± SD; 95% confidence interval 2.7 to 4.8%) and 4.4 ± 4.7% (mean ± SD; 95% confidence interval 3.4 to 5.5%), respectively. Cartilage volume in each individual seemed to track over the study period, relative to other study participants. After multivariate adjustment, annual medial tibial cartilage loss was predicted by lesser severity of baseline knee pain but was independent of age, body mass index and structural factors. No factors specified *a priori *were associated with lateral cartilage volume rates of change. Tibial cartilage declines at an average rate of 4% per year in subjects with symptomatic knee OA. There was evidence to support the concept that tracking occurs in OA. This may enable the prediction of cartilage change in an individual. The only significant factor affecting the loss of medial tibial cartilage was baseline knee pain, possibly through altered joint loading.

## Introduction

Clinicians, faced with a patient with osteoarthritis (OA), have taken a somewhat nihilistic approach with respect to the modification of structural disease progression. Modifiable risk factors for disease progression have been difficult to identify with radiographic measures; the possible exception is weight loss, although evidence to support this is inconsistent [1,2]. Our understanding of joint cartilage development and the pathophysiology of OA has previously been limited by the lack of a non-invasive method for assessing joint cartilage *in vivo*. There has been increasing interest in the use of magnetic resonance imaging (MRI) to measure the disease severity of knee OA [3-6].

Knee cartilage volume measured with MRI is one such approach, which shows promise as a method of quantifying disease severity in OA. It is a valid and reproducible measure of articular cartilage [5,7]. It correlates inversely with radiographic grade of disease, such that subjects with knee OA have less knee cartilage than normal healthy subjects [8]. It is possible to estimate normal cartilage volume to distinguish diseased knees from healthy ones [8,9].

Once knee OA is established, knee cartilage tends to be lost more rapidly than in healthy adults [10-13]. Over 2 years, we have shown the annual rate of loss of total tibial cartilage to be between 4.4% and 6.2% in people with symptomatic knee OA [10], nearly double the rate of loss in healthy subjects without knee pain [12,13]. Although it has been suggested that cartilage loss is episodic in OA, structural evidence to support this is lacking [14]: it is unclear whether the average rate of loss is stable. Complicating this is the recognised high variability of cartilage loss both between individuals who are healthy or who have OA [10,12] and between those with progressive and non-progressive OA [14]. It is therefore unclear whether the average rate of loss remains similar over the longer term or the pattern of loss is linear.

Studies with only two measures are unable to examine patterns of change and are limited in their capacity to examine for potential risk factors for disease progression because change is confounded with measurement error. In addition, regression to the mean will induce a spurious negative correlation between initial cartilage volume and change in cartilage volume. Longitudinal studies with more than two measures for each subject have the potential to provide a better estimate of the true change for each subject than do studies with two measures, because a true underlying linear change can be distinguished from measurement error and other sources of within-individual variability over time [15]. We have extended the observation of a cohort of community-dwelling subjects with predominantly mild symptomatic knee OA to determine the change in knee cartilage volume in subjects with knee OA over 4.5 years [10], and factors that may affect this.

## Materials and methods

This report is an extension of the observation (at an average of 4.5 years) of a community-based cohort of 123 subjects with symptomatic mild knee OA, who had previously been followed for 2 years to determine the rate of cartilage loss [10]. All participants in the previous study who had undergone baseline MRI, who were alive, who had not received a joint replacement in the study joint and who had no contraindication to MRI imaging (such as a pacemaker, a metal implant or claustrophobia) were invited to take part in this study. There were 105 eligible subjects since 18 of the original participants had undergone knee replacement surgery.

Subjects with mild to moderate knee OA had been recruited by advertising, as described previously [10]. The study was approved by the ethics committee of the Alfred and Caulfield Hospitals in Melbourne, Australia. All subjects gave informed consent.

### Inclusion criteria

Inclusion criteria were age over 40 years and symptomatic (at least one pain dimension of the WOMAC (Western Ontario and McMaster University Osteoarthritis Index) score above 20% and osteophytes present) knee OA (American College of Rheumatology clinical and radiographic criteria [16]). Subjects were excluded if any other form of arthritis was present or if there was a contraindication to MRI (such as a pacemaker, a cerebral aneurysm clip, a cochlear implant, the presence of shrapnel in strategic locations, metal in the eye, or claustrophobia), inability to walk 50 feet without the use of assistive devices, hemiparesis of either lower limb, or planned total knee replacement.

At baseline (time zero), each subject had a weight-bearing anteroposterior tibiofemoral radiograph, taken in full extension, of the symptomatic knee. Where both knees had OA and were symptomatic, the knee with the least severe radiographic OA was identified and used. These were independently scored by two trained observers who used a published atlas to classify disease in the tibiofemoral joint. The radiological features of tibiofemoral OA were graded in each compartment, on a four-point scale (0 to 3) for individual features of osteophytes and joint space narrowing [17]. In the event of disagreement between observers, the films were reviewed with a third independent observer. Intra-observer and inter-observer reproducibility for agreement on features of OA (osteophytes and joint space narrowing, grades 0 and 1 versus grades 2 and 3) ranged between 0.85 and 0.93 (κ statistic) [18].

At baseline (time zero) and at each subsequent visit (2 and 5 years), subjects were weighed to the nearest 0.1 kg (after removal of shoes and bulky clothing) with a single pair of electronic scales, and their height was measured to the nearest 0.1 cm (shoes removed) with a stadiometer. Body mass index (BMI; weight/height^2 ^(kg/m^2^)) was calculated. General health status was assessed with the Short Form 36 [19]. Knee function (0 to 1,700), pain (0 to 500) and stiffness (0 to 200) were assessed with the WOMAC at baseline, 2 years and 5 years, where 0 represents no symptoms [20].

### MRI assessment

An MRI was performed on each subject's symptomatic knee (or the knee with the least severe radiographic OA where both were symptomatic) at baseline, two years later [10] and about five years later (present study). Knee cartilage volume was determined by image processing on an independent workstation with the software program OSIRIS, as described previously [5,18]. Knees were imaged in the sagittal plane on the same 1.5-tesla whole-body magnetic resonance unit (Signa Advantage HiSpeed GE Medical Systems, Milwaukee, WI, USA) as used previously, using a commercial receive-only extremity coil. The same sequence and parameters were used as in the previous study [10]. Sagittal images were obtained at a partition thickness of 1.5 mm and an in-plane resolution of 0.31 mm × 0.83 mm (512 pixels × 192 pixels).

Cartilage volume was measured at time 0 and 2 years by two trained observers, and the data were used to compare baseline and loss to follow-up. For examination of change in knee cartilage over 5 years, all MRI taken at 0, 2 and 5 years were remeasured by two different trained observers. The volume of cartilage overlying osteophytes was not included in measurements. Measurements of all MRI on a single subject were made within one month, independently, blinded to subject identification and timing of MRI. Each of the two observers measured cartilage volume on each scan once. Their results were compared. If the results were within ± 20%, an average of the results was used. If they were outside this range, the measurements were repeated until the independent measurements were within ± 20%, and the averages used. Repeat measurements were made blind to the results of the comparison of the results of the other scans. The coefficients of variation for the measurement of total, medial and lateral cartilage volume measures were 2.6%, 3.4% and 2.0%, respectively [18].

Areas of medial and lateral tibial plateaux were determined by creating an isotropic volume from the input images, which were reformatted in the axial plane. Areas were measured directly from these images. The coefficients of variation for the measurement of the medial and lateral tibial plateau areas were 2.3% and 2.4%, respectively; the average of the areas was used [18]. Osteophytes were not included in these measurements.

Knee angles were measured by a single observer, as has previously been described from standing anteroposterior radiographs [21-23]. Lines were drawn through the middle of the femoral shaft and through the middle of the tibial shaft. The angle subtended at the point at which these lines met in the centre of the tibial spines was based on a modified method of Moreland and colleagues [21], as described and used recently [22,23]. The angle subtended by the lines on the medial side was measured with OSIRIS software. Thus, an angle less than 180° was more varus and an angle greater than 180° more valgus. The intra-observer variability in 50 subjects 4 weeks apart was 0.98 (intraclass correlation coefficient).

### Statistical analysis

Descriptive statistics for characteristics of the subjects were tabulated. Independent-samples *t *tests and χ^2 ^tests were used to compare variables in those who completed the study with those who were lost to follow-up. Annual change in cartilage volume was computed as (initial volume minus second volume)/time between scans, so that positive numbers reflect a loss of cartilage. Annual percentage change in cartilage volume was computed as 100 × (initial volume minus second volume)/(initial volume multiplied by time between scans). The difference in average rate of change over the two study periods (0 to 2 years and 2 to 5 years) was assessed by paired *t *tests. To explore the possible factors affecting the rate of change in cartilage volume, random coefficient models were employed [15]. Conceptually, these models are formulated via a two-stage process. In the first stage, a linear regression model is postulated for the true underlying pattern of change of cartilage volume over time for each individual (for instance, initial cartilage volume and rate of change), and these patterns are allowed to differ for each individual. In the second stage, regression models are postulated to ascertain how the baseline and rate of change of cartilage volume parameters from the first-stage model vary according to specified factors/covariates [15]. The factors we considered in such analyses were age, gender, height, weight, BMI, baseline WOMAC scores (pain, stiffness and function), initial cartilage volume, bone size, grade of osteophyte present, and knee malalignment. Models with a linear rate of change over time for each individual were applied, together with main effect and interaction terms with time for each potential predictive factor. Assessments of model assumptions were made by means of residual diagnostic plots [15]. Analyses were performed with the SPSS statistical package (version 12.0.1; SPSS, Cary, NC, USA), and with Stata (version 9; Stata Corporation, College Station, TX, USA) for the random coefficient modelling.

## Results

One hundred and five subjects were eligible for this study. Of these, 78 (74%) subjects completed the study, by undergoing a third MRI scan at 4.5 ± 0.35 years (mean ± SD). Reasons for failure to participate included significant co-morbidity (9), moved interstate (3) and loss to follow-up/refusal to participate (15). Those subjects who were unable to complete the study because of total knee joint replacement before the third MRI were ineligible to participate.

The demographic and baseline characteristics of the subjects are shown in Table 1. Subjects who failed to complete the final follow-up were compared with those who completed the study. The annual percentage medial and lateral tibial cartilage loss in the first time period was similar in those who completed the study and in those who did not.

The raw data, using only two time points, suggested that medial tibial cartilage and lateral tibial cartilage increased in 13 and 7 people, respectively, over the course of the study. However, using the random coefficient model, incorporating all three measures for each subject to improve the estimate of individuals' true underlying rates of change together with 95% prediction intervals, showed that only one of these subjects exhibited an increase beyond the prediction uncertainty for lateral volume only, thereby suggesting a possible true increase in lateral cartilage volume for this individual.

**Figure 1:**
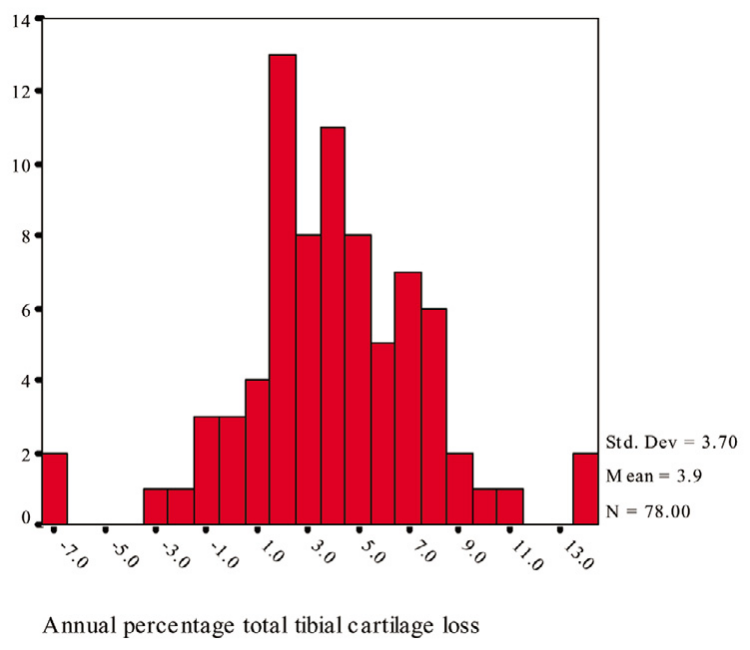
Individual annual percentage rate of total tibial cartilage loss.

Over 4.5 years, the average amount of 'total' tibial cartilage (medial plus lateral tibial cartilage) lost per year was 135 ± 135 mm^3^/year (Table 2). When this was calculated as a percentage of the initial baseline cartilage, this represented an annual rate of loss of 'total' tibial cartilage of 3.9 ± 3.7% (mean ± SD; 95% confidence interval (CI) 3.1 to 4.8%). The distribution of the annual percentage change in total cartilage is shown in Figure 1. The average amounts of medial and lateral tibial cartilage lost per year were 62.7 ± 78 mm^3 ^and 72.2 ± 73 mm^3 ^(Table 2). This represents an annual rate of loss of medial tibial cartilage of 3.7 ± 4.7% (mean ± SD; 95% CI 2.7 to 4.8%) of initial cartilage. Lateral tibial cartilage was lost at an annual rate of 4.4 ± 4.7% (mean ± SD; 95% CI 3.4 to 5.5%) of initial cartilage. Over the complete period, the annual volume of loss of medial and lateral tibial cartilage was moderately correlated (*r *= 0.60, *p *< 0.001). There was evidence of tracking of both medial and lateral cartilage volumes over time, in that the relative rankings of cartilage volumes of individuals remained similar across the three time periods of the study (Spearman rank correlations ranged from 0.67 to 0.89 for medial, and from 0.87 to 0.94 for lateral).

Factors affecting the rate of annual medial and lateral tibial cartilage loss over the whole study period are shown in Table 3, in which both univariate and multivariate adjusted associations are presented. A sample interpretation of the results is as follows: for the medial compartment, the average rate of loss in males was estimated as 28.3 μm^3^/year greater than the rate of loss of females (*p *= 0.08), and the difference in the average rate of loss for people who differed by 10 years in their age at initial measurement was 1.6 μm^3^/year (*p *= 0.84). Examining the remainder of the univariate analyses in Table 3, annual rates of medial tibial cartilage loss were significantly increased in those with higher initial medial tibial cartilage volumes and greater bone area, and reduced in those with more severe knee pain initially (WOMAC pain score). Only the effect of initial knee pain persisted after multivariate adjustment. Although no other factors were shown to affect change in the lateral compartment significantly in either univariate or multivariate analyses, the direction of effect and magnitude of effects were generally similar to those observed in the medial compartment.

Because pain was the only important factor in predicting cartilage loss, and it may be understood to comprise the separate domains of biomechanical and inflammatory disorders, we performed a post hoc analysis. In this, we grouped the three mechanical questions within the pain subscale (pain walking on the flat, pain walking up/down stairs, pain standing upright) and the two questions relating to inflammation (pain in bed at night and pain sitting/lying up) together to examine the effects of these domains on change in medial and lateral cartilage volume. These demonstrated that increased symptoms captured by the biomechanical questions were related to reduced cartilage loss in both medial (*p *= 0.03) and lateral (*p *= 0.01) cartilages. However, the questions on inflammation were not related to change in cartilage volume (*p *= 0.22 medial, *p *= 0.07 lateral).

There was no evidence that the change in cartilage volume over time was nonlinear (*p *= 0.50 for medial and *p *= 0.62 for lateral quadratic time effects). Analysis of residual diagnostic plots did not display any evidence of violation of the assumption of linearity of the change over time for individuals. Additional analyses (not tabulated) relating change in knee pain and change in weight to rate of cartilage loss did not produce any significant results, nor did examination of level of physical activity, knee angle or grade of osteophyte.

**Figure 2:**
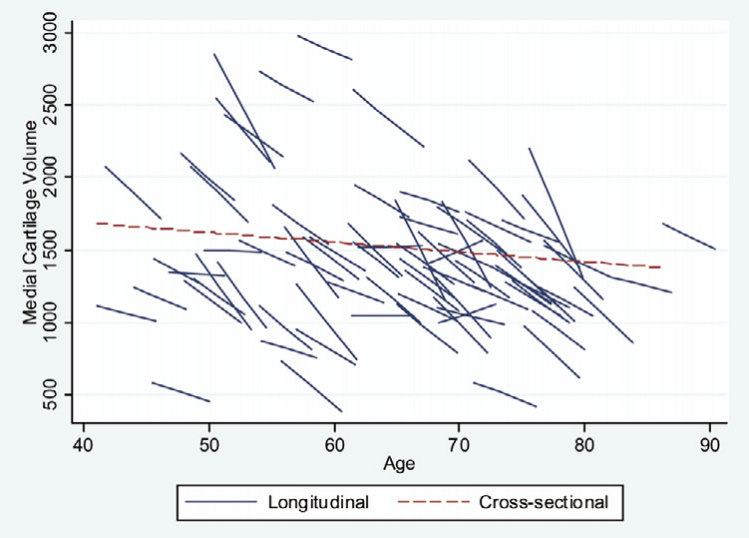
Predicted effects of ageing on medial cartilage volume using a random coefficient model.

We examined whether the estimate of change in cartilage volume within an individual over time (the longitudinal effect of ageing) was similar to the estimate of change with age, using data that compared different people of different ages (the cross-sectional effect of ageing). To assess this, we fitted a random-effects model with initial age and time since initial measurement as covariates. Figure 2 shows the predicted medial cartilage volume changes for individuals as they aged over the study period (such as longitudinal effects), together with the regression line obtained with only each individual's initial age (for instance, cross-sectional effect of age). The regression model estimated that medial tibial cartilage volume is lost at an average rate of 61.2 μm^3^/year (*p *< 0.001). The difference in cartilage volume of two individuals whose age differed by 1 year would be expected to be 6.6 μm^3 ^(*p *= 0.17). The pseudo-*R*^2 ^value for the amount of within-individual variation explained by a linear time effect was 56% [15]. Results for lateral cartilage were similar although less marked (within-individual annual change, 71.2 μm^3^, *p *< 0.001, versus cross-sectional difference 14.2 μm^3^, *p *= 0.012, pseudo-*R*^2 ^= 66%).

## Discussion

We showed that in 78 subjects with symptomatic knee OA, the rate of tibial cartilage loss was ongoing beyond 2 years, at a rate of between 3.1% and 4.8% per year over 4.5 years. Higher initial pain scores were associated with diminished medial and total tibial cartilage loss. There was a moderately strong positive correlation between the percentage loss of medial and lateral tibial cartilage. In addition, the use of the random coefficient modelling structure enabled an assessment of the relationship between the true rate of cartilage loss and initial cartilage volume – an assessment that is plagued by regression to the mean when using only the observed rates of change and initial cartilage volumes in the sample. For example, the finding for the medial compartment that the relationship between initial volume and rate of change was diminished after multivariate adjustment would not have been obtained if the simple observed rates of change had been regressed on the observed initial volumes, and a spurious effect of initial volume as a predictor of change would have emerged.

This is the first study to examine whether average annual cartilage loss in subjects with symptomatic knee OA is similar over the medium term, with biannual loss stable over two periods. We found the average total tibial cartilage loss to lie between 3.1% and 4.8% per year over 4.5 years. These results are consistent with the magnitude of loss observed in previous studies that examined subjects over 2 years [10,11,24]. Over 2 years, in the initial cohort of 123 subjects with symptomatic knee OA, we showed the annual rate of loss of total tibial cartilage to lie between 4.4% and 6.2% [10]. Over 24 months, another group, examining 32 subjects, found similar results, with the annual total tibial cartilage loss between 2.2% and 6.6% [11]. The only study examining change in 11 subjects over about 3 years found no mean significant change over this time period [24]. However, the 95% confidence intervals for annual medial tibial cartilage change included a loss of 3.9% per year to a gain of 3.0% per year in that study. We found that the distribution of rate of change was normal over 2 years, and also at 4.5 years [10]. The sensitivity to change of the MRI volume measurements over the various periods using the Standardized Response Mean index [25] can be obtained simply from Table 2 by dividing the average change by the SD of the changes. These are of the order of 0.50, 0.75 and 0.90 for the periods 0 to 2 years, 2 to 5 years and 0 to 5 years, indicating that MRI volume measures display increased sensitivity to change with longer follow-up of subjects.

The moderately strong correlations seen between cartilage volume measured at each time point suggests that tracking of individual change in cartilage volume occurs in those with knee OA. This means that subjects maintain their relative ranking over time in terms of cartilage volume, compared with other subjects within the study. This phenomenon has been described in adult women aged 30 to 94 years, regarding tracking of bone mineral density [26]. It has also been shown to occur in children and adolescents with regard to height and also to the accrual of bone mineral content [27]. It has been suggested that this is under genetic control, unless strong environmental factors intervene. Similarly, change in cartilage volume has also been shown to have strong genetic determinants in healthy adult children of those who have had knee arthroplasties [28].

Our results for predicted rates of change for individuals suggested that only one subject gained lateral cartilage volume beyond prediction uncertainty over the course of the study. It is possible that this increase in volume was related to a gain of cartilage or it might have been due to swelling related to early OA in that compartment [29,30]. Quantification of cartilage volume is unable to differentiate between these two possibilities.

Some cross-sectional studies have suggested an effect of age on cartilage volume [8,9], although others have not [31]. Only a longitudinal study, such as this, is able to differentiate between the within-individual cartilage loss of individuals (for instance, expected change in one person over one year) as they age and the cross-sectional effects of age across different subgroups of people (for instance, comparisons between people who differ by one year of age only). The average rate of medial cartilage loss within an individual over 1 year was estimated as 61.2 μm^3^/year (longitudinal effect of age), whereas the difference in average volumes of two groups of people differing by 1 year in age (cross-sectional effect of age) is expected to be 6.6 μm^3 ^(Figure 2). The longitudinal rate of loss in lateral tibial cartilage was 71.3 μm^3^/year compared with the cross-sectional difference of 14.2 μm^3 ^per year of age. This illustrates that, in this study population, the longitudinal rates of change are far greater than cross-sectional differences, which is similar to findings relating to bone mineral density [32,33]. This suggests that interpreting cross-sectional age effects in studies with similar populations as representing change in an individual may be unhelpful and misleading, The implication is that classification systems based on these assumptions may be flawed.

There is increasing evidence that biomechanical effects are important in the progression of knee OA [34-36]. We found lower levels of knee pain to be associated with higher rates of cartilage loss in the medial tibial cartilage. This contrasts to previous findings, with our initial report of this cohort (123 subjects) [10] and a similar study (110 subjects) [37], both over 2 years, of no effect of baseline pain over 2 years. The present study took place over 4.5 years and showed that for every increase in WOMAC pain score of 10 (of a maximum of 500), there was an reduction in annual cartilage loss of about 10% (6.7 mm^3^/year). The previous studies, over a shorter period, may not have had power to show this effect. Our findings may be explained by subjects with painful knee OA adapting their gait in response to pain, to reduce pain, by reducing external adduction moment [38,39]. This reduces compressive force on the cartilage, which may reduce cartilage loss [1]. Conversely, pain reduction increases external adduction moment, which has been associated with increased disease progression [34,38]. Joint loading has been shown to affect articular cartilage in knee OA [34].

The main study limitation relates to the loss to follow-up of 27 subjects, with a completion rate of 74%. The subjects who did not undergo knee joint replacement surgery and were lost to follow-up were similar to those who completed the study, including disease severity, and lost cartilage at a similar rate during the first 2 years of the study. Indeed, of the 123 subjects who participated in the initial study, 63% underwent MRI at the 4.5 years follow-up. The 18 who underwent knee joint replacement have already been shown to have lost cartilage more rapidly than those who did not undergo joint replacement [40]. It is possible that our study might have underestimated somewhat the average rate of cartilage loss in all subjects with OA, given the exclusion of subjects who underwent knee replacement over the study period, who are known to lose cartilage more rapidly [40]. The presence of swelling in early OA may also reduce the cartilage loss seen.

We found few predictors of rate of change in cartilage volume. This may be because there is a strong genetic component to cartilage loss [28] or may simply reflect a lack of power related to limited sample size, reducing our ability to detect modest determinants of change. Other factors such as meniscal pathology and cruciate ligament integrity have been shown to affect the incidence of OA and subsequent cartilage loss [41,42]. We were unable to examine for these pathologies because of the limited MRI sequences that were used in this study.

Another limitation is that the model was restricted to assuming that change was linear because only three time points were available. This may be a reasonable assumption over a short period (less than five years) because we found no evidence to the contrary. However, the assessment of linearity in this study was complicated by the large amount of variability observed in cartilage volume measurements for individuals around their predicted change. Observing a greater number of measurements over time will be needed to confirm whether the pattern of cartilage loss is linear. The within-individual variability in cartilage volumes not accounted for by linear loss may be due to other factors that may affect change in cartilage volume varying with time, such as effusion, trauma, inflammatory change and pain. It may also be that loss is not truly linear, in contrast with reduction in bone mineral density [26], but shows mild exponential 'decay', with reduced activity. Finally, despite our attempts to minimise measurement error by means of reader training and blinding, it is possible that this still represents a substantial component of the unexplained within-individual variability.

## Conclusion

OA is a complex disease, affected by many factors, including genetic, environmental, traumatic and biomechanical factors. This study shows that change in knee OA occurs in a linear pattern and provides an estimate of cartilage loss over the medium term (4.5 years), which will help in study design and also in estimating the effect of interventions aimed at reducing structural change in knee OA. Larger studies, incorporating multiple determinants identified in the pathogenesis of OA, may be able to extract the relative roles of these varied factors. This study cautions against the use of cross-sectional data to make statements about longitudinal changes with age within individuals.

## Abbreviations

BMI = body mass index; CI = confidence interval; MRI = magnetic resonance imaging; OA = osteoarthritis; WOMAC = Western Ontario and McMaster University Osteoarthritis Index.

## Competing interests

The authors declare that they have no competing interests.

## Authors' contributions

AEW coordinated the study, data measurement, initial analysis and interpretation of data and drafting of the manuscript. AF performed the statistical analysis and was involved in manuscript review. YY and FH were involved in data measurement and manuscript review. GJ provided intellectual content for the manuscript and was involved in manuscript review. FMC was involved in study conception, supervision of the group and manuscript preparation. All authors read and approved the final manuscript.

## Figures and Tables

**Table 1:** Characteristics of study population

Characteristic	Eligible population (*n *= 105)	Subjects with MRI at 4.5 years (*n *= 78)	Subjects lost to follow-up at 4.5 years (*n *= 27)	*p*^a^
Age, years	62.5 (10.7)	63.8 (10.6)	61.6 (11.3)	0.65
Number of women, %	59 (53%)	44 (54%)	17 (63%)	0.50
Height, cm	168 (9.3)	168 (9.2)	167 (9.5)	0.45
Weight, kg	80.1 (15.0)	80.5 (15.3)	79.3 (14.4)	0.72
BMI	28.4 (5.1)	28.4 (4.8)	28.7 (5.9)	0.79
Womac				
Pain	79 (45)	78 (43)	81 (53)	0.82
Stiffness	38 (23)	37 (21)	42 (28)	0.39
Function	298 (171)	290 (161)	324 (201)	0.47
Total	416 (228)	406 (214)	449 (272)	0.49
Kellgren Lawrence^b^				
I	11	7	4	
II	48	35	13	
III	45	35	10	0.10
Knee angle^c^, degrees	181 (6)	181 (6)	180 (5)	0.38
Tibial plateau area, mm^2^				
Medial	2,065 (405)	2,085 (394)	2,008 (438)	0.42
Lateral	1,358 (257)	1,352 (239)	1,374 (308)	0.73
Cartilage volume at baseline, mm^3^				
Medial	1,754 (500)	1,780 (513)	1,676 (459)	0.33
Lateral	1,945 (580)	1,946 (576)	1,946 (602)	0.98
Annual cartilage loss over first period, %				
Medial	4.3 (6.3)	4.3 (6.5)	4.3 (7.4)	0.87
Lateral	4.7 (6.8)	4.1 (5.9)	6.1 (4.6)	0.15

Results are reported as mean (SD), except where variables are categorical. Body mass index (BMI) is measured as weight in kilograms divided by the square of height in metres; WOMAC, Western Ontario and McMaster University Osteoarthritis Index. ^a^The *p *value for difference between subjects who had undergone baseline magnetic resonance imaging (MRI) and had subsequently completed 5-year MRI and those who did not complete 5-year follow-up. Comparisons made with Student's *t *test or χ^2^, for categorical variables, or Eta test for categorical variable by nominal. ^b^One X-ray was lost after study began. ^c^Angles available for 96 subjects.

**Table 2:** Rate of change for subjects completing follow-up, over the total, first and second time periods

Parameter	4.5 years^a^	First period: 0–2 years^a^	Second period: 2–5 years^a^	Difference (95% CI)
Annual rate of cartilage loss, mm^3^/year				
Total	135 (135)	143 (247)	125 (144)	18 (- 46 to 81)
Medial	63 (78)	69 (149)	56 (85)	12 (- 28 to 52)
Lateral	72 (73)	75 (140)	69 (77)	6 (- 31 to 42)
Annual percentage cartilage loss				
Total	3.94 (3.68)	4.04 (7.25)	4.11 (4.39)	- 0.07 (- 1.91 to 1.76)
Medial	3.74 (4.72)	3.87 (9.49)	3.77 (5.88)	0.10 (- 2.44 to 2.64)
Lateral	4.42 (4.65)	4.60 (9.10)	4.80 (5.60)	- 0.20 (- 2.40 to 1.99)

CI, confidence interval. ^a^Mean (SD).

**Table 3:** Factors potentially affecting change in cartilage volume over 4.5 years

Factor	Univariate analysis		Multivariate analysis^a^		
	Regression coefficient	*p*	Regression coefficient	95% CI	*p*
Medial cartilage					
Initial age^b^	1.6	0.84	3.7	- 10.9 to 18.2	0.62
Gender^c^	28.3	0.08	12.6	- 29 to 54	0.55
Height^d^	1.11	0.20	-	-	-
Weight^e^	- 0.65	0.90	-	-	-
Body mass index^f^	- 1.68	0.34	1.1	- 2.3 to 4.6	0.53
Initial cartilage volume^g^	41.2	0.15	18.3	- 19.4 to 56.1	0.34
Bone size^h^	4.61	0.03	3.4	- 1.8 to 8.6	0.21
WOMAC pain^i^	- 6.7	<0.001	- 7.1	- 10.8 to - 3.4	<0.001
Lateral cartilage					
Initial age^b^	3.7	0.63	4.0	- 11.2 to 19.3	0.61
Gender^c^	22.9	0.15	7.2	- 36.3 to 50.7	0.75
Height^d^	1.2	0.14	-	-	-
Weight^e^	1.9	0.72	-	-	-
Body mass index^f^	- 0.8	0.65	0.2	- 3.4 to 3.9	0.90
Initial cartilage volume^g^	- 1.2	0.93	- 23.6	- 63.2 to 16.0	0.24
Bone size^h^	3.6	0.07	2.9	- 2.6 to 8.4	0.29
WOMAC pain^i^	- 1.6	0.40	- 1.7	- 5.5 to 2.2	0.40

BMI, body mass index; CI, confidence interval; WOMAC, Western Ontario and McMaster University Osteoarthritis Index. ^a^Multivariate analysis with age, gender, BMI, initial cartilage volume, bone size and initial pain (WOMAC) score in regression equation; ^b^difference in rate of loss of cartilage volume per 10-year difference in age; ^c^difference in rate of loss of cartilage volume for males compared with females; ^d^difference in rate of loss of cartilage volume per centimetre difference in height; ^e^difference in rate of loss of cartilage volume per 10 kg difference in weight; ^f^difference in rate of loss of cartilage volume per unit increase in BMI; ^g^difference in rate of loss of cartilage volume per 1,000 mm^3 ^increase in initial cartilage volume; ^h^difference in rate of loss of cartilage volume per 100 mm^2 ^increase in bone area; ^i^difference in rate of loss of cartilage volume per 10-unit increase in baseline WOMAC pain score.
